# The costs of interdigital phlegmon in four loose-housed Finnish dairy herds

**DOI:** 10.1186/s13028-015-0181-4

**Published:** 2015-12-30

**Authors:** Johanna Häggman, Reijo Junni, Heli Simojoki, Jarmo Juga, Timo Soveri

**Affiliations:** Department of Agricultural Sciences, University of Helsinki, FIN-00014 Helsinki, Finland; Environmental Health office of Central Ostrobothnia, Vasarakuja 15, FIN-67100 Kokkola, Finland; Department of Production Animal Medicine, University of Helsinki, FIN-00014 Helsinki, Finland

**Keywords:** Dairy, Interdigital phlegmon, Footrot, Economics

## Abstract

**Background:**

The aim of the study was to provide detailed herd level cost information about an outbreak of interdigital phlegmon (IP), which has been an emerging problem with enlarged loose house barns in Finland in recent years. During enlargement, the farmer’s financial situation is sensitive because of the large investments to the farm business and unexpected costs can risk the farm’s survival.

**Results:**

The University of Helsinki research herd and three commercial herds having outbreaks of IP in 2012 or 2013 were visited to collect detailed information about the costs and economic impact of the outbreaks. The majority of the costs came from the discarded milk due to the antibiotic treatments. In Finland IP is usually treated with parental benzylpenicillin for 5 days which result in discarded milk for a total of 11 days. Third generation cephalosporins, widely used in other countries, have no milk withdrawal time. However, the use of these antibiotics is not recommended in Finland since these antimicrobials are critically important for human health. Herd-level costs varied between 4560 and 28,386 € depending on the herd size, the frequency of the infected cows, the antibiotics used and other costs involved. The average cost per infected cow was 489 €.

**Conclusions:**

The outbreaks of IP cause severe economic losses to dairy farms and the costs are lower if cows are treated with antibiotics with no withdrawal time. However, other costs, such as involuntary culling, reduced production and fertility also produce substantial costs to the farms. Early detection of sick animals, rapid treatment and control measures to limit the outbreak of IP can lower the costs. Because of the high costs farms should concentrate on preventing the disease.

## Findings

Interdigital phlegmon (IP) or foot rot is a necrotizing soft tissue inflammation in the interdigital region of the foot [1–3]. IP is believed to be a mixed infection of multiple anaerobic pathogens, such as *Fusobacterium necrophorum* (which is believed to be the primary pathogen), *Dichelobacter nodus*, *Porphyromonas levii, P. asaccharolytica, Prevotella intermedia,* and *P. melaninogenica* [3, 4]. However, the exact pathogenic mechanisms of these bacteria are still not determined. *F. necrophorum* is commonly found in the soil and can be isolated on the feet, rumen and feces of healthy cows [3], hence injury to the skin of the interdigital area can predispose the cow to IP [2]. In recent years, outbreaks of IP have occurred in Finnish dairy farms. IP is painful, causes lameness, and is also a costly disease, which can have a serious impact on the farm profitability. Specifically, the effect of the disease is dramatic if the majority of the cows in a herd are infected.

In Finland IP is usually treated with parenteral benzylpenicillin for 5 days (20 mg/kg) with a milk withdrawal time of 6 days, which results in discarded milk of a total of 11 days. Additionally, cows are usually treated with non-steroidal anti-inflammatory drugs and in some cases foot baths for the healthy cows are recommended for controlling the infection in the herd. If IP is diagnosed and treated early the antibiotic treatment is usually successful and most cases respond rapidly [1], whereas the response to delayed antibiotic treatment can fail to control the infection and in the worst case the cow has to be culled [5]. Permanent working group on the antimicrobials of the Ministry of Agriculture and Forestry in Finland has set guidelines for antimicrobial treatments of common diseases in animals. The primary antimicrobial recommendation for IP is use of narrow-spectrum antibiotic benzylpenicillin and the secondary use of oxytetracycline or macrolides [6]. Ceftiofur, a third generation cephalosporin, does not have a milk withdrawal time and is available in Finland. However, as it belongs to the group of antimicrobials, which is identified by the World Health Organization (WHO) as critically important for human health, the use of this antimicrobial is not recommended since its impact to antimicrobial resistance [7]. According to the one health perspective, Nordic countries have managed to keep the prevalence of antibiotic resistant at a low level which is important to maintain the effectiveness of these antibiotics also in the future. If the effectiveness of these antibiotics is lost there will be a need to develop a new medicine which is turned out to be challenging.

The aim of this study was to provide detailed cost information about an outbreak of IP at the herd level. Such information is not possible to obtain from the standard Finland health or economic recording and no previous studies have been undertaken to compile the economic costs associated with an IP outbreak.

The costs presented in this study do not include taxes (VAT 0 %). In the University research farm the accurate daily discarded milk yields were available. In the other farms losses due to the discarded milk were calculated based on the number of the clinical cases of IP, the milk yield (*my*) in the previous test day recording before the clinical case of IP was diagnosed, the wait time (*wt*) due to antibiotic use and the milk price (*mp*) at that time.

$$Milk = \sum\limits_{i = 0}^{n} {\left( {my \times wt \times mp} \right)}$$

The veterinary costs were calculated based on the number of veterinarian visits and the costs of treatment per clinical case including all veterinary fees (*vf*) and medicines (*m*).

$$Veterinarian = \sum\limits_{i = 0}^{n} {\left( {vf + m} \right)}$$

The labor costs were calculated based on time spent (*ts*) on the clinical case and the hourly wage claim (*wc*) of the dairy farmer in 2013 in Finland (14.90 €) [8]. The estimated daily time spent for a clinical case was 15 min/cow for farms A, C and D and 30 min/cow for farm B.

$$Labor = \sum\limits_{i = 0}^{n} {\left( {ts \times wc} \right)}$$

The costs for special claw trimmings were calculated based on the number of the claw trimmings due to clinical cases and the rate of the trimming (*rt*).

$$Trimming = \sum\limits_{i = 0}^{n} {rt}$$

The cost of an involuntary culled cows and lost calves were calculated based on the total number of culled cows and lost calves, the transportation cost (*tc*), the price of the culled cows (*pc*) which varied between 500 and 1250 € depending on the parity, milk yield and fertility status and the price of the lost calves (*plc*) which was 100 €/calf.

$$Culled\_animals = \sum\limits_{i = 0}^{n} {\left( {tc + pc + plc} \right)}.$$

Other costs, such as the increased use of copper sulfate in the foot baths, were also included. IP can also lower the milk production, prolong the calving interval and predispose cows to other diseases [5]. A decrease in milk production was not noted in this study. The information about the cost of the prolonged calving interval was not available and was not included in this study. However, an average dairy cow with a clinical foot disorder has a prolonged calving interval of 12 days [9] and in Finland the daily cost for each open day is about 2.5 € [10].

University of Helsinki research herd (A) and three commercial loose-housed herds (B–D) having outbreaks of IP in 2012 or 2013 were visited to collect detailed information about the costs and economic impact of the outbreak. These farms had not had IP outbreaks before and the previous status of other infectious claw disorders was comparable to other farms in Finland. The commercial farms had already participated in the “Infectious hoof diseases in dairy cows in new loose housing system” project funded by the Ministry of Agriculture and Forestry. The researcher visited the farms at the beginning of the outbreaks and diagnosed IP based on clinical signs (body temperature, appearance and site of the lesion, swelling, odour, lameness) and bacteriology. Later IP cases at the farm were diagnosed by the local veterinarian. Farm location, number of cows, number of cows treated and antimicrobials used are described in Table 1 and the cost sources are described in Table 2.

**Table 1:** Farm location, number of cows, number of cows treated and antimicrobials used

Farm, location	Cows total (n)	Infected cows (n)	Antimicrobials used during IP outbreak
A, Southern Finland	65	50	71 treatments: benzylpenicillin^a^ (300,000 IU/ml)
			1 treatment: oxytetracycline^b^ (100 mg/ml)
B, Central Finland	40	7	4 treatments: oxytetracycline^b^ (100 mg/ml)
			6 treatments: benzylpenicillin^c^ (300 mg/ml)
C, Northern Ostrobothnia	80	36	22 treatments: benzylpenicillin natrium^d^ (24 g)
			18 treatments: benzylpenicillin^c^ (300 mg/ml)
			4 treatments: benzylpenicillin^a^ (300,000 IU/ml)
			11 treatments: ceftiofur^e^ (50 mg/ml)
D, Central Finland	85	68	6 treatments: oxytetracycline^b^ (100 mg/ml)
			32 treatments: benzylpenicillin^c^ (300 mg/ml)
			37 treatments ceftiofur^e^ (50 mg/ml)

^a^Ethacillin vet, MSD Animal Health, Finland, dose 20,000 IU/kg once a day 5 days

^b^Engemycin LA vet, MSD Animal Health, Finland, dose 10 mg/kg 5 days

^c^Penovet vet, Vetcare, Finland, dose 20 mg/kg once a day 5 days

^d^Geepenil vet, Orion, Finland, dose 10 mg/kg, twice a day 4 days

^e^Cefenil vet, Vetmedic Animal Health OY, Finland, dose 1 mg/kg, once a day 3 days

**Table 2:** The percentage of infected cows, herd-level costs (€) and costs divided with the number of infected cows

Farm	A	B	C	D
Infected cows (%)	77	18	45	80
Cost due to discarded milk	19,989.1	1702.2	6135.5	5865.7
Veterinary cost^a^	7056.1	1133.0	5000.0	6621.3
Extra labor cost	1341.0	678.0	1385.7	1825.3
Extra claw trimming cost		1047.0	500.0	1371.5
Costs due to culling			3820.0	
Other costs			900.0	1033.0
Total cost	28,386.2	4560.2	17,741.2	16,716.6
Total cost/infected cow	567.7	651.5	492.8	245.8

^a^cost of medicines included

The costs varied between the farms (Table 2), herd-level costs being the highest in the University research farm (A), where the costs due to the discarded milk were high when the majority of the cows were infected, milk production was high and cows were treated with regular antibiotics. In two study herds (C and D), however, cows were treated with ceftiofur (Table 1) and milk losses were not severe as some veterinarians use it despite the recommendations. The average cost per infected cow was 489 €, the lowest costs in farm D where the majority of the cows were infected and half of them were treated with ceftiofur. In farm B only a small number of cows were infected and the total costs were low which could be due to the early detection of the disease and the fact that affected cows were isolated from the rest of the herd during the early infectious stages. All the cows in farm B were monitored carefully and closely inspected if something abnormal was observed. In farm C, seven calves were lost and four cows had to be culled producing extra costs.

IP epidemics cause economic losses to dairy industry and the majority of the costs come from the discarded milk due to the treatments with antibiotics. Also, other costs involved (involuntary culling, reduced production and fertility) are substantial. However, rapid treatment and control measures to limit the outbreak of IP will greatly reduce the total herd cost. Based on this study the average cost per infected cow can be assumed to be 600–700 € when the cow is treated with benzylpenicillin. Because of high costs of IP it would be more efficient on the economic point of view to focus on the prevention of the disease.
